# The natural compound Jatrophone interferes with Wnt/β-catenin signaling and inhibits proliferation and EMT in human triple-negative breast cancer

**DOI:** 10.1371/journal.pone.0189864

**Published:** 2017-12-27

**Authors:** Iram Fatima, Ikbale El-Ayachi, Ling Taotao, M. Angeles Lillo, Raya Krutilina, Tiffany N. Seagroves, Tomasz W. Radaszkiewicz, Miroslav Hutnan, Vitezslav Bryja, Susan A. Krum, Fatima Rivas, Gustavo A. Miranda-Carboni

**Affiliations:** 1 Department of Medicine, College of Medicine at UTHSC, UTHSC Center for Cancer Research Memphis, Tennessee, United States of America; 2 Department of Chemical Biology and Therapeutics, St. Jude Children's Research Hospital, Memphis, Tennessee, United States of America; 3 Department of Orthopaedic Surgery and Biomedical Engineering, UTHSC, Center for Cancer Research, UTHSC, Memphis, Tennessee, United States of America; 4 Department of Pathology and Laboratory Medicine, College of Medicine at UTHSC, UTHSC Center for Cancer Research Memphis, Tennessee, United States of America; 5 Department of Experimental Biology, Faculty of Science, Masaryk University, Brno, Czech Republic; University of Alabama at Birmingham, UNITED STATES

## Abstract

Metastatic breast cancer is the leading cause of worldwide cancer-related deaths among women. Triple negative breast cancers (TNBC) are highly metastatic and are devoid of estrogen receptor (ER), progesterone receptor (PR) and human epidermal growth factor receptor 2 (HER2) amplification. TNBCs are unresponsive to Herceptin and/or anti-estrogen therapies and too often become highly chemoresistant when exposed to standard chemotherapy. TNBCs frequently metastasize to the lung and brain. We have previously shown that TNBCs are active for oncogenic *Wnt10b/β-catenin* signaling and that WNT10B ligand and its downstream target HMGA2 are predictive of poorer outcomes and are strongly associated with chemoresistant TNBC metastatic disease. In search of new chemicals to target the oncogenic WNT10B/β-CATENIN/HMGA2 signaling axis, the anti-proliferative activity of the diterpene Jatrophone (JA), derived from the plant *Jatropha isabelli*, was tested on TNBC cells. JA interfered with the WNT TOPFLASH reporter at the level between receptor complex and β-catenin activation. JA efficacy was determined in various subtypes of TNBC conventional cell lines or in TNBC cell lines derived from TNBC PDX tumors. The differential IC_50_ (DCI_50_) responsiveness was compared among the TNBC models based on etiological-subtype and their cellular chemoresistance status. Elevated *WNT10B* expression also coincided with increased resistance to JA exposure in several metastatic cell lines. JA interfered with cell cycle progression, and induced loss of expression of the canonical Wnt-direct targets genes AXIN2, HMGA2, MYC, PCNA and CCND1. Mechanistically, JA reduced steady-state, non-phosphorylated (activated) β-catenin protein levels, but not total β-catenin levels. JA also caused the loss of expression of key EMT markers and significantly impaired wound healing in scratch assays, suggesting a direct role for JA inhibiting migration of TNBC cells. These results indicate that Jatrophone could be a powerful new chemotherapeutic agent against highly chemoresistant triple negative breast cancers by targeting the oncogenic *Wnt10b/β-catenin* signaling pathway.

## Introduction

Breast cancer is one of the most common non-cutaneous malignancies among women, and each year it afflicts approximately 1.5–2.2 million women worldwide (World Health Organization, WHO). In the United States breast cancer is a leading cause of death in women, with greater than 40,000 deaths per year. Many of these deaths are due to rapid onset of chemoresistant disease in triple negative breast cancer (TNBC) cases, which are devoid of estrogen receptor (ERα^-^), progesterone receptor (PR^-^) and human epidermal growth factor receptor (HER2-) amplification. TNBC’s poor overall prognosis reflects its propensity to metastasize to visceral organs throughout the body combined with the lack of targeted therapies to treat the disease [1].

Targeted therapies for ERα^+^ breast cancer include tamoxifen and aromatase inhibitors. Patients diagnosed with HER2^+^ breast cancers receive the monoclonal antibody Herceptin to treat this subtype, which has been in the clinical use for well over 20 years. In contrast, TNBCs do not have specific-targeted therapeutics, are high-grade tumors with poor prognosis, and are highly metastatic. Moreover, TNBC patients who rely on standard neoadjuvant chemotherapeutics, for example, doxorubicin and or cyclophosphamide, often become chemoresistant within three years of diagnosis [2]. If TNBC arise from the BRCA carrier mutations (~5% of TNBC), then the therapeutic regimen has been one of several PARP-inhibitors (such as veliparib), which have failed as single agents, but have shown some promise when combined with carboplatin and/or paclitaxel after neoadjuvant chemotherapy [3].

*Wnt/β-catenin* signaling is activated by interaction of WNT-ligands with their co-receptors, subsequently leading to the stabilization of non-phosphorylated *β*-catenin, often referred to as activated β-catenin (ABC). WNT ligands stimulate inactivation of a multi-protein “destruction complex” consisting of tumor suppressor adenomatous polyposis coli (APC), AXIN1, two Ser/Thr kinases, CK1 and GSK-3β, and the ubiquitin ligase β-TrCP [4]. Stabilized *β*-catenin translocates to the nucleus, interacting with TCF/LEF and induces specific transcriptional programs controlling several cellular responses including, but not limited to, cellular proliferation, development, differentiation, neoplasia and stem cell maintenance [5]. TNBCs are highly heterogeneous, composed of at least 6 subtypes [6], and *Wnt/β-catenin* signaling is known to be activated in the basal-like 2 (BL2), mesenchymal-like (ML), and mesenchymal stem-like (MSL) subtypes, which are the most difficult subtypes of TNBC to treat. We have shown that expression of the Wnt ligand, WNT10B, and the WNT10B downstream target, HMGA2, predict poor survival (both genes) and metastasis (HMGA2 alone) in women with BL2, ML, and MSL TNBC [7]. We have shown that the WNT10B/β-CATENIN/HMGA2 axis is expressed in the majority of metastatic TNBC cases (metTNBC) derived from women of either African-American (AA) or European American (EA) descent [6].

Natural products (NPs) have provided a direct source of therapeutic agents and a basis for drug development for the past 60 years [8]. Nature provides unique structural architectures that can lead to new therapeutic agents. As part of our collaborative efforts to identify new chemical entities against cancer using a high throughput NP fractionation system for a Hit-to-Lead drug discovery platform, we conducted a phenotypic cell-based screen using a small library of natural product fractions and pure natural products (12K entities) using an established CellTiterGlo cell viability assay [9,10]. The library included fractions and pure natural products from terrestrial plants from the American continent with recorded ethnopharmacological properties [9]. Several hits were identified against leukemia cellular models and also against Raji Non-Hodgkin Lymphoma. The Raji model carries an activated Wnt/*β*-catenin program and is highly metastatic [11], features also observed in TNBC. Here, we focus on one of those hit compounds, namely jatrophone (JA). JA Jatrophone is a macrocyclic diterpene featuring a unique oxaspiro core and several electrophilic centers. JA was isolated from the *Jatropha isabelli* and *Jatropha gossypiifolia* plants belonging to the Euphorbiaceae family. JA displays a broad range of biological properties, including antitumor, cytotoxic, anti-inflammatory, anti-malarial and fungicidal properties [12]. Although, JA’s specific biological target(s) are elusive and remain undefined, its potent biological activity warrants further study.

Herein, a secondary-screen utilizing the Wnt-reporter TOPFLASH system, identified that JA interferes with Wnt/β-catenin signaling somewhere between the receptor and β-catenin activation. We exposed various chemoresistant TNBC cell lines or cell lines derived from treatment-naïve or treatment refractory TNBC PDX models to JA and determined the IC_50_ concentrations. We provide evidence that JA exerts its anti-proliferative properties in TNBC cells *via* interference with the canonical *Wnt/β-catenin* signaling pathway, and subsequently inhibits the expression of Wnt-direct downstream targets. The potent anti-proliferative activity of this compound in TNBC cells demonstrated that JA could serve as a lead candidate molecule for development of an efficacious anti-breast cancer agent for the management of disease in patients diagnosed with highly chemoresistant metastatic TNBC.

## Material and methods

### Compound

**Fig 1:**
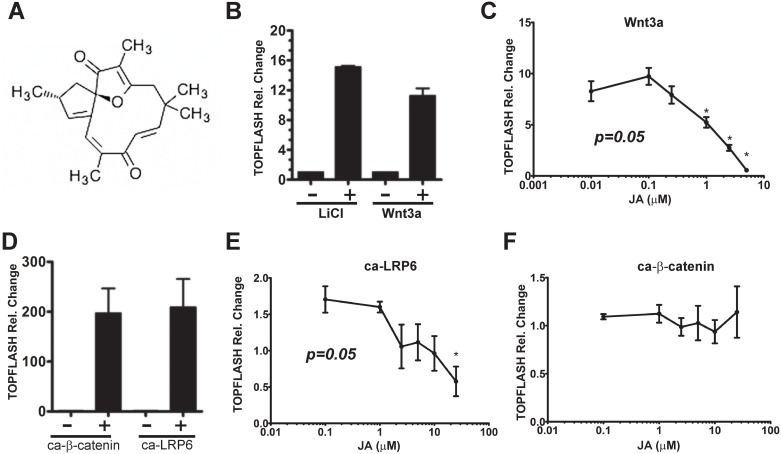
Compound JA interferes with Wnt/β-catenin. **A)** Structure of Jatrophone (JA). **B)** LiCl (25μM for 24 hours) and WNT3A activate Super 8xTOPFLASH Wnt reporter system in HEK293T cells. **C)** JA inhibits WNT3A activation of TOPFLASH. **D)** Constitutively active (ca) ΔLRP6 and ca β-catenin robustly active TOPFLASH Wnt reporter system in 293T cells. **E)** JA is capable of inhibiting Wnt reporter activity by ca ΔLRP6 in a concentration dependent manner but not ca β-catenin **F)**. The firefly luciferase activity was normalized to *Renilla* luciferase activity. Following concentrations of JA were used: 0.1, 1, 2.5, 5, 10, 25 μM for both ca-LRP6 and ca-β-catenin. Concentration of JA for inhibiting WNT3A were 10 nM, 100 nM, 250 nM, 1 μM, 2 μM and 5 μM. The experiment in C and D was performed in four biological replicates, *p < 0.05, One-way Anova, Tukey´s post test.

Jatrophone (JA) (Fig 1A) was isolated from 31 *Jatropha gossypiifolia* (Florida, USA) roots and stems (100 g), which were extracted in a Soxhlet apparatus in refluxing ethanol (3 x 500 mL) for 48 h. The mixture was filtered and the solvent was evaporated to afford the crude mixture as dark green syrup, which was purified by normal-phase silica gel column chromatography (5%-50% EtOAc/Hex). The non-polar compounds obtained were re-purified via normal-phase silica gel column chromatography (5%-15% EtOAc/Hex). Several unknown compounds were identified along with 5 mg jatrophone (**1**, 0.5% by mass) and large quantities of cyperenoic acid. JA was confirmed by 1D, 2D NMR experiments and mass spectroscopy. These data were identical to JA isolated from *Jatropha isabelli* (Paraguay).Jatrophone (**1**) White solid; ^1^H NMR (400 MHz, methanol-*d*_4_) δ 6.58 (d, *J* = 16.3 Hz, 1H), 5.96 (d, *J* = 16.3 Hz, 1H), 5.79–5.69 (m, 2H), 3.02 (d, *J* = 15.1 Hz, 1H), 2.98–2.89 (m, 1H), 2.49 (d, *J* = 15.1 Hz, 1H), 2.18 (dd, *J* = 13.7, 5.9 Hz, 1H), 1.85 (s, 3H), 1.78 (dd, *J* = 13.7, 7.7 Hz, 1H), 1.71 (s, 3H), 1.36 (s, 3H), 1.24 (s, 3H), 1.08 (d, *J* = 7.0 Hz, 3H); ^13^C NMR (100 MHz, CD_3_OD) δ 206.30, 204.14, 186.98, 162.44, 148.39, 143.92, 138.80, 129.48, 124.57, 113.9 1, 101.51, 43.50, 42.05, 39.78, 38.04, 30.53, 27.23, 20.97, 19.69, 6.04; HRMS (ESI-TOF) calculated for C_20_H_24_O_3_ ([M + H]^+^): 313.1803, found: 313.1805.

### Reporter gene assay

The activity of β-catenin dependent Wnt signaling was measured by the TOPFLASH reporter using the Dual Luciferase Reporter Assay (E1960, Promega, Madison, WI) according to the manufacturer’s instructions. Briefly, HEK-293T cells were grown in 24-well plates and cells were transfected with 0.1 μg of Super8X TOPFLASH plasmid (gift from Randall Moon), 0.1 μg of pTK-Renilla luciferase (Promega) and 0.3 μg of the constitutively active (ca) LRP6 receptor (Lrp6ΔN, pCS2+VSV-G Lrp6ΔN, a gift from Xi He) or ca-β-catenin, using a polyethylenimine transfection protocol described elsewhere[13]. Six hours after transfection, the transfection medium was replaced with DMEM-Hi supplemented with 10% FBS overnight in the presence or absence of the compound JA. At 24 h post-transfection, cells were lysed with commercial reporter lysis buffer (Promega), and luminescence was measured by the Promega Dual-Luciferase Reporter System according to the manufacturer’s protocol on a Hidex Plate Chameleon V plate reader. Firefly luciferase signal was normalized to Renilla luciferase expression and data expressed in relative luciferase units (RLU), n = 4 biological replicates. For the Wnt3a ligand-dependent reporter assays, HEK-293Tcells were transfected with the pcDNA-Wnt3A-V5 plasmid (Addgene [14]) using Lipofectamine 3000 (Invitrogen, Carlsbad, CA) per the manufacturer’s protocol. To normalize transfection efficiency in the reporter assays, cells were co-transfected with 0.1 μg of the internal control reporter, pTK-Renilla luciferase. Sixteen hours after transfection, the culture medium was replaced by DMEM-Hi supplemented with 10% FBS in the presence or absence of compound JA. Twenty-four hours after transfection, cells washed with PBS, lysed and luciferase activities in cell lysates measured as described above. The experiments performed in three biological replicates, with the results normalized as described above.

### Cell culture, cell synchronization and proliferation assay

Human triple negative breast cancer cell lines MDA-MB-231, HCC38, MDA-MB-157 and MDAMB-468, human osteosarcoma cell lines, U2OS and MG3, and the HeLa cervical cancer cell line were maintained in DMEM/RPMI plus 1% Pen/Strep and 10% fetal calf serum (FCS) in a humidified atmosphere with 5% CO_2_. All cell lines were purchased from ATCC (Manassas, VA) and have been authenticated by Genetica by STR profiling (https://www.celllineauthentication.com). For the PDX TNBC models, stably passaged cell lines were derived freshly digested HCI-2 (TNBC, treatment-naïve) or HCI-10 (TNBC, treatment refractory) tumors that had been passaged in Nod/Scid/Gamma mice and the isolated tumor epithelial cells were cultured in monolayer in M87 complete medium using published protocols [15], generating HCI-2 cells (cHCI-2) and HCI-10 cells (cHCI-10). HCI-2 cells were maintained in a humidified atmosphere with 5% CO_2_ in DMEM/F12 supplemented with 2% FBS, 1x insulin-transferrin-selenium (ITS), 1x penicillin-streptomycin-glutamine, human epidermal growth factor (5 ng/mL), hydrocortisone (0.3 μg/mL), cholera toxin (0.5 ng/mL) 3,3’,5-triiodo-L-thyronine (5 nM), isoproterenol hydrochloride (5 μM), ethanolamine (50 nM) and *O*-phosphorylethanolamine (50 nM). HCI-10 cells were maintained in a humidified atmosphere with 5% CO_2_ in DMEM/F12 supplemented with 2% FBS, 1x insulin-transferrin-selenium (ITS), 1x penicillin-streptomycin-glutamine, human epidermal growth factor (5 ng/mL), hydrocortisone (0.3 μg/ml), cholera toxin (0.5 ng/mL) 3,3’,5-triiodo-L-thyronine (5 nM), isoproterenol hydrochloride (5 μM), ethanolamine (50 nM), *O*-phosphorylethanolamine (50 nM) and HEPES (10 mM). The synchronization of cell lines at G_1_ to evaluate cell cycle progression is described elsewhere [7]. ICG-001 (in 1% DMSO) and JA (in 1% DMSO) were used in experiments with synchronized cell as follows: treatment began 16 hours post-release from G_1_, i.e. progression into S-phase, for an additional 48 hours. Cell proliferation was measured using the WST-1 cell proliferation assay (Roche, Basel, Switzerland) according to the manufacturer’s protocol.

### Annexin-V/propidium iodide (PI) labeling and flow cytometry assay for apoptosis

Annexin-V binding is indicative of early apoptosis. MDA-MB-231 (2×10^5^cells/ml) were cultured in six-well plates treated with 2 μM JA for 48 h, harvested and washed with phosphate-buffered saline (PBS). Both the adherent and non-adherent cell fractions were probed with fluorescein isothiocyanate (FITC)-conjugated Annexin-V and PI (Sigma, St. Louis, MO) for 15 min. The staining profiles were determined with FACScan and Cell-Quest software. The experiments were performed two times.

### Cell cycle analysis

MDA-MB-231 cells were seeded (2 × 10^5^ cells/ml) into 6-well plates and treated with either 10μM ICG-001 or 2μM JA for 48 h. After treatment, cells were washed with PBS, fixed in chilled 75% ethanol, treated with RNase and then stained with PI solution (50 μg/ml). Cell cycle distribution was analyzed with the FACSCalibur analyzer (BD Biosciences, Franklin Lakes, NJ) and Cell-Quest software. The percentage of DNA content at different phases of the cell cycle was analyzed with Modfit-software (Verity Software House, ME, USA).

### RNA and real-time PCR

Isolation of total RNA was performed using TRIzol (Life Technologies) according to manufacturer’s protocol. RNA was treated with DNA-free kit (Ambion, Austin, TX) and converted to cDNA with Maxima First Strand cDNA Synthesis Kit (Fermentas, Waltham, MA) according to the manufacturer’s protocol. cDNA was subjected to quantitative PCR (qPCR) using the Step One Plus (Applied Biosystems) or Realplex2 cycler (Eppendorf, Hamburg, Germany). qPCR was conducted in a final volume of 20 μl using the Maxima SYBR Green/ROX qPCR Master Mix (Fermentas) according to the manufacturer's protocols. Amplification conditions were: 95°C (5 s), 40 cycles of 95°C (30 s), 55°C (60 s) and 72°C (60 s). Primer pairs for each gene are provided in Supplemental S1 Table.

### Wound healing assay

Cells (2,500 cells/200 μl/well) were seeded in a 96-well plate and incubated at 37°C until 90% to 100% confluent. Confluent cells were scratched with an 8-channel mechanical “wounder”, followed by washing with PBS, and cells were then treated with JA in complete medium. After 16 h of incubation, the cells were fixed and stained with 2% ethanol containing 0.2% crystal violet powder for 15 min, and randomly chosen fields were photographed under a light microscope using a 43x objective. The mean number of cells migrated into the scratched area was then calculated per condition. IncuCyte S3^®^ Live-Cell Analysis System by Essen BioScience was used to capture the video for 48hrs; the illustrated video was for 16 hours only.

### Cell extraction and western blotting

Cells were lysed as previously described [16] using RIPA buffer (#9806, Cell Signaling Technology, Danvers, MA) supplemented with protease inhibitor cocktail (#5871, Cell Signaling Technology) and 1 mM PMSF (#195381, MP Biomedicals, LLC, Burlingame, CA). Western analysis was conducted by resolving 10–50μg of protein per lane using 8–10% polyacrylamide SDS-PAGE gels. After transferring to Immobilon-P membrane (Millipore, Temecula, CA) the membrane was blocked with 5% skimmed milk and then incubated with each appropriate primary antibody overnight at 4°C. The following primary antibodies were used: Myc (#SC-764), Cyclin D1 (#SC-718) and Pan β-catenin (#SC-7199) antibodies, purchased from Santa Cruz Biotechnology (Santa Cruz, CA). Antibodies for Axin2 (#ab32197) were purchased from Abcam (Cambridge, MA). Actin (#3700), non-phosphorylated (Active) β-catenin (#8814), PCNA (#2586), HMGA2 (#5269) and GSK3β-Ser9 (#9336) were procured from Cell Signaling Technology. Membranes were then washed and incubated with ImmunoPure-peroxidase conjugated secondary antibodies (ThermoScientific, Waltham, MA) for 1 h. Antibody binding was detected using SuperSignal West Pico plus enhanced chemiluminescent (ECL) detection system (#34577, ThermoScientific). After developing, the membrane was stripped (#21059, ThermoScientific) and the membranes re-probed using another primary antibody of interest, or β-actin to confirm equal loading.

### Immunofluorescence (IF)

For IF assays, cells were cultured in 8-well chamber slides (ThermoScientific) and fixed with 4% paraformaldehyde-PBS for 15', washed, incubated with blocking buffer (TBS pH 7.8, 3% BSA, 1% normal goat serum, 1% Triton X-100, 0.01% NaAzide) and stained. Immunofluorescence was conducted with non-phosphorylated (Active) β-Catenin (#8814, Cell Signaling), followed by incubation with a goat anti-rabbit-IgG-Alexa Fluor 594 antibody (Life Technologies, 1/250, #A11037). DAPI was used to visualize nuclei (Vector labs). Digital images were captured using the EVOS FLO Cell Imaging System and software (Life Technologies) and or the Spectrum Database for Whole Slide Imaging System (Perkin Elmer).

### Statistical methods

For all the experiments, statistical analysis was performed using Prism 5 software (Graph Pad, San Diego, CA) including two-tailed Student’s *t*-test and one-way ANOVA analysis. All graphs were generated using either Microsoft Excel or Prism 5 software. All error bars are represented as the mean ± SEM.

## Results

### Jatrophone inhibits the TOPFLASH Wnt-reporter

WNT ligand interaction with the LDL-related protein 6 (LRP6) initiates signal transduction, which results in the inactivation of the β-catenin destruction complex that includes adenomatous polyposis coli (APC), AXIN1 and glycogen synthase kinase-3β (GSK-3β). Inactivation of GSK-3β prevents cytoplasmic β-catenin phosphorylation and concurrently the accumulation of β-catenin levels, its nuclear translocation and downstream target gene activation.

To study the influence of JA (Fig 1A) on Wnt activity, we utilized plasmids encoding either the WNT3A ligand (i.e. pcDNA-WNT3A-V5 Addgene [14]), constitutively active (ca)-LRP6 (ΔN-LRP6) and ca-β-catenin (S33A-β–catenin) [13]. WNT3A ligand functions upstream at the receptor level, whereas ca-LRP6 bypasses the requirement for WNT ligand, but acts upstream of β-catenin, and ca-β-catenin cannot be phosphorylated by GSK-3β, and, therefore, is independent of the upstream destruction complex events (mediated by AXIN1/APC/GSK3β). Lithium chloride (LiCl at 20 μM), is a known direct inhibitor of GSK-3β, [17] and has been used to induced Wnt/β-catenin signaling gene induction [18]. LiCl can robustly activate the Wnt specific-reporter, TOPFLASH in HEK-293T cells after a 24 hour treatment (Fig 1B). Concurrently, WNT3A is capable of inducing TOPFLASH activation in those same cells. To determine if the NP JA has inhibitory effects at the ligand-level, HEK-293T cells were co-treated with increasing concentrations of JA (10 nM, 100 nM, 250 nM, 1 μM, 2 μM and 5 μM) after transfection of the WNT3A plasmid (Fig 1C). JA significantly decreases TOPFLASH reporter activation at the three highest concentrations of JA (*p = 0*.*05*). Moreover, in the absence of JA, both ca-LRP6 and ca-β-catenin also robustly activated the TOPFLASH reporter in HEK-293T cells (Fig 1D). To gain further insights on JA inhibitory activity on TOPFLASH signaling at either the membrane level, or in the nucleus, we co-treated HEK-293T cells transfection with either ca-LRP6 or ca-β-catenin with increasing JA concentrations (0.1 μM, 1 μM, 2.5 μM, 5 μM, 10 μM to 50 μM) as shown in Fig 1E & 1F. Whereas repression of the ca-LRP6 activation of TOPFLASH activity is JA concentration-dependent (Fig 1E), JA failed to block reporter activation induced by ca-β-catenin (Fig 1F). These data demonstrate that JA can inhibit Wnt/β-catenin signaling at the level between receptor complex and nuclear β-catenin, suggesting JA-dependent interference of Wnt activity occurs early in the Wnt signaling cascade.

### JA impairs the growth of triple negative breast cancer cells

**Fig 2:**
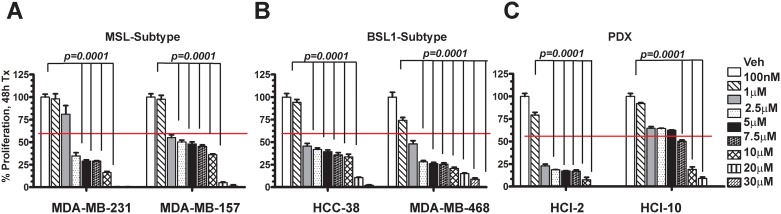
Determination of differential IC_50_ (DIC_50_) effects of JA on various TNBC cell lines and TNBC PDX-derived cell lines. WST-1 proliferation assays for 48 hours utilizing JA at various dosages ranging from 100 nM-30 μM: **(A)** MSL-subtype TNBC cell lines MDA-MB-231 (EA) vs. MDA-MB-157 (AA). **(B)** BL1-subtype TNBC cell lines HCC-38 (EA) vs. MDA-MB-468 (AA). **(C)** TNBC PDX-derived “naïve” cell line HCl-2 vs. “chemoresistant” HCl-10. The IC_50_ of JA indicated for each cell lines determined by n = 3, in triplicate, using the two-tailed *t-test p*-values were calculated: *p < 0.05, **-p < 0.01 *** < 0.001 relative to vehicle control cells (DMSO).

TNBC is a very heterogeneous disease that has been identified to have at least six molecular subtypes: immunomodulatory (IM), mesenchymal (M), mesenchymal stem-like, basal-like 1 and 2 (BL1 and BL2) and luminal androgen receptor (LAR) [6]. Each of these TNBC subtypes has its own unique ontology, subsequently, they respond differently to standard of care (SOC) treatments [19]. To determine if JA induces differential responses among TNBC cellular subtypes, the MSL and BL1 subsets were tested. The MSL subtypes MSL (MDA-MB-231, EA vs. MDA-MB-157, AA) are shown in Fig 2A and the BL1 subtypes (HCC38, EA vs. MDA-MB-468, AA) are depicted in Fig 2B. To study anti-cancer potential of JA, WST-1 assays (Roche) were conducted using JA dosages ranging from 100 nM-30 μM for 48 hours. JA treatment led to statistically significant (*p = 0*.*0001*) decreases in cellular proliferation in both MSL subtype cells relative to vehicle control (DMSO). The IC_50_ value for MDA-MB-231 cells was ~2.0 μM, with less than 30% viability observed at dosages ranging from 2.5–7.5 μM. At higher dosages (10–30 μM), less than 2% of the cells were viable. In contrast, the IC_50_ concentration of MDA-MB-157 cells was ~3.5 μM with at least ~40% of the cells viable between 7.5–10μM. Similar to the MDA-MB-231 cells, the MDA-MB-157 cells were not viable (<2%) at the two highest dosages used. We repeated the JA exposure in the two BSL1 TNBC subtype cell lines HCC38 (EA) and MDA-MB-468 (AA) (Fig 2B). Treatment with JA reveals that both BSL1 subtype HCC38 and MDA-MB-468 cells have similar IC_50_ values of ~2μM and 1μM respectively. Moreover, HCC38 cells are statistically significant less responsive than the MDA-MB-468 cells at doses ranging from 2.5–10 μM concentration relative to vehicle alone (*p = 0*.*0001*).

Breast cancer research employing patient-derived xenograft (PDX) tumors generated by transplanting primary human tumor samples into immunocompromised NOD/SCID/Gamma (NSG) mice has provided an advance over cell line-based xenograft modeling [20]. We obtained two TNBC PDX breast cancer models from the Huntsman Cancer Institute (from Dr. Alana Welm). It should be noted that the HCI-2 TNBC PDX tumor was derived from a treatment-naïve (i.e. never received chemotherapy) patient and that the HCI-10 TNBC PDX was derived from a chemoresistant patient (i.e. over 2 years of SOC-therapy). We established PDX TNBC cell lines from each of these models as previously described [15], to determine their response to JA exposure (Fig 2C). HCI-2 (naïve) TNBC cells respond robustly to JA exposure; at a dose of 1 μM there was only ~25% viability. In contrast, HCI-10 TNBC cells do not respond robustly to JA, with an IC_50_ of ~6.5 μM; moreover, they maintained ~10% of cell viability at doses up to 20 μM. We compared the IC_50_ values for all TNBC models (S1A Fig) and determined that MDA-MB-157 and HCI-10 cells had the highest IC_50_ values, indicating they are less sensitive to JA. Next, we hypothesized that the various responses to JA exposure may be related to differential expression of WNT10B ligand among these models, which would subsequently lead to higher levels of transcriptionally activated β-catenin signaling that could account for the higher IC_50_ values observed for both the MDA-MB-157 and HCI-10 cells (S1A Fig).

We confirmed the above hypothesis as both the MDA-MB-157 and HCI-10 cells were observed to have the highest levels of WNT10B expression (S1B Fig). To further support this notion we analyzed the expression of *WNT10B* in HeLa cells (a cervical cancer cell line), which is known to have the highest expression of *WNT10B* within the NIH’s NCI-60 cell line panel (data not shown), and in U2OS cells (osteosarcoma), which are known to express *WNT10B; WNT10B* expression is associated with chemoresistance in these cell lines [21]. We confirmed elevated levels of *WNT10B* mRNA expression in both HeLa and U2OS cells and determined a higher resistance to JA therapy in those same cells (S1C and S1D Fig). In contrast, MG-63 cells (another osteosarcoma cell line) had no detectable expression of *WNT10B* mRNA, similar to either MCF-7 (ER+) and MCF-10A (a TNBC control “normal” cell line), and MG-63 cells responded robustly to JA exposure as compared to U2OS cells (S1D Fig).

In summary, JA interferes with TNBC cell proliferation at differential IC_50_ values based on the cancer cell line etiology-subtype, the known cellular chemoresistance status and/or associations with elevated mRNA levels for *WNT10B*.

### JA induces apoptosis and inhibits proliferation in S-phase more effectively than the classic WNT inhibitor ICG-001

**Fig 3:**
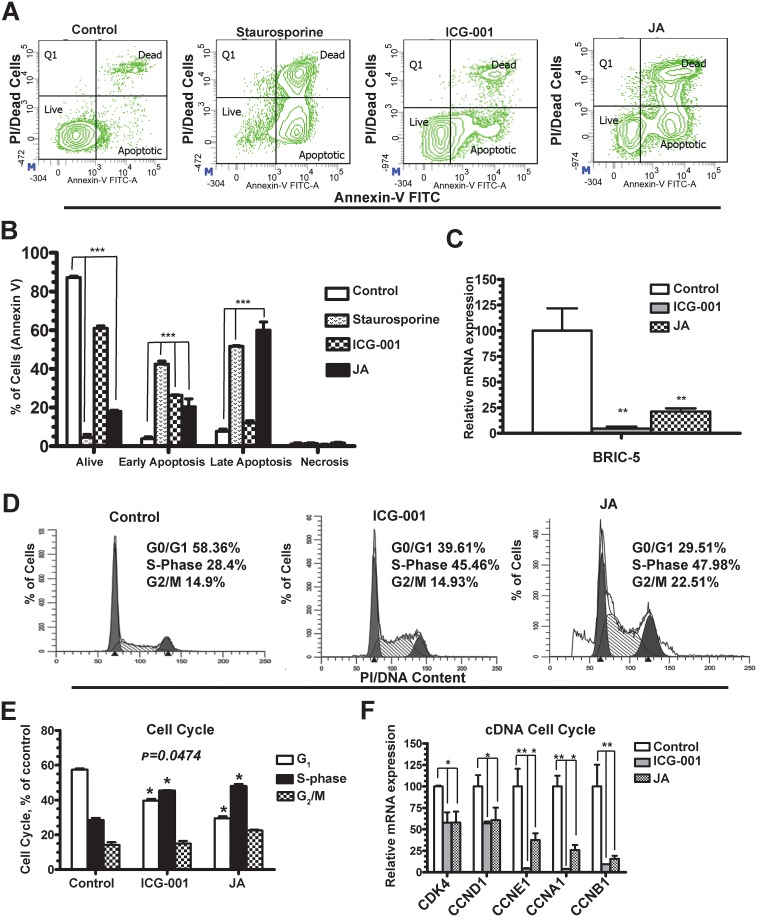
Compounds JA arrests cells at S-phase of cell cycle and induced apoptosis in MDA-MB-231 Cells. **A)** Annexin V-FITC staining was used to detect apoptosis by flow analysis in control (DMSO), staurosporine (1μM), ICG-001 (10 μM) and JA (2.5 μM) treated cells for 48 hours. Cells were counter stain with propidium iodide (PI, 1 μg/ml). We show one representative FACS plot **B)** Quantification of alive, early and late apoptotic cells in cells from panel A. **C)** qPCR for Survivin (*BIRC5*) in control cells, ICG-001 and JA treated cells. **(D)** One representative FACS plot using PI/DNA content analyzed by flow cytometry **(E)** Quantification of cell cycle phases for in G_0_/G_1_, S and G_2_/M in both ICG-001 and JA treated cells. **(F)** qPCR for cell cycle markers *CDK4*, *CCND1*, *CCNE1*, *CCNA1 and CCNB1* from the same cells as in panel E. Statistics on three biological independent experiments with duplicates for each of the above. *T-test* was used to determine *p*-values: ***-p < 0.001, **-p < 0.01 and *p < 0.05 vs. control.

To investigate the mechanism of action of JA on the highly chemoresistant TNBC MDA-MB-231 MSL-subtype we determined its effects on both apoptosis (Fig 3A–3C) and proliferation (Fig 3D–3F), utilizing ANNEXIN V and propidium iodide (PI)/DNA content labeling in conjunction with flow cytometry. We have previously shown that the WNT pathway inhibitor ICG-001 (i.e. a specific CBP/β-catenin antagonist) at 10 μM can block CBP/β-catenin-mediated proliferation of MDA-MB-231 cells, which are dependent on both WNT10B and its downstream target HMGA2 [7]. ICG-001 is also known to induce apoptosis *via* a survivin-dependent mechanism [22], and serves as our control for both proliferation and apoptosis assays. JA (2 μM) was able to elicit late apoptotic events more effectively than ICG-001 (~60% vs. ~12%; *p* = 0.001) whereas the early apoptotic events were similar for both drugs (~20% vs. 26%), relative to control cells treated with either DMSO vehicle or staurosporine (1 μM) (Fig 3A and 3B). We confirmed the loss of survivin (*BIRC5*) mRNA expression by qPCR (Fig 3C). These results suggest that JA is inducing apoptosis through a similar pathway as ICG-001.

We next studied the effect of JA on cell cycle distribution. The experiments were conducted as previously published in the MDA-MB-231 cell line exposed to ICG-001 at 10 μM for 48h [7]. In brief, the cells were synchronized by contact inhibition at the G_0_/G_1_ phase of the cell cycle prior to the exposure of cytotoxic agents for a duration of 16 hours after release from synchronization, followed by analysis with flow cytometry using PI to measure DNA content (Fig 3D). JA affects G_2_/M accumulation more robustly than ICG-001 (i.e. 22.51% vs. 14.93%), however each drug was equally effective in arresting cells at the S-phase of the cell cycle relative to control cells (i.e. 47.98%, JA vs. 45.46%, ICG-001; n = 3 biological replicates of triplicate technical replicates, *p* = 0.041). These results demonstrate that JA is statistically significant at blocking both the S- and G_2_/M phases of the cell cycle relative to control cells (Fig 3E).

Cell cycle progression is governed by both cyclin-dependent kinases (CDKs) and various cyclins regulating cell cycle progression (i.e. G_0_/G_1_, S and G_2_/M). Both CDKs and cyclins coordinate the timing and progression through each of the four phases of the cell cycle. Furthermore, this process is uniquely regulated by *Wnt10b/β-catenin* signaling in mammary tumors *in vivo* and in mouse mammary epithelial cell lines *in vitro* [16]. Additionally, we have previously shown that WNT10B has epistatic activity on HMGA2 and its downstream targets CCNA1 and/or CCNB1, which is necessary and sufficient for proliferation of TNBC cells [7]. Thus, WNT10B and its downstream target HMGA2 strongly coordinated cell cycle progression *in vivo* and *in vitro* in MDA-MB-231 cells, which could be inhibited by both ICG-001 and the Porcupine inhibitor Wnt-C59, which interferes with secretion of all WNT ligands [7]. To determine which of the cell cycle regulators are responsible for JA-dependent effects, we conducted qPCR for *CDK4* & *CCND1* (G_1_ to S-phase), *CCNA1* & *CCNE1* (late G_1_ to S-phase) and *CCNB1* (G_2_/M transition) mRNA expression (Fig 3F). JA significantly reduced the expression of all of the cell cycle regulators, similar to ICG-001 treatments. Overall, JA was more effective at inducing apoptosis and cell cycle arrest as compared to ICG-001 in the highly chemoresistant TNBC MDA-MB-231 cell line.

### JA inhibited non-phosphorylated activated *β*-catenin (ABC) signaling in MDA-MB-231 cells

**Fig 4:**
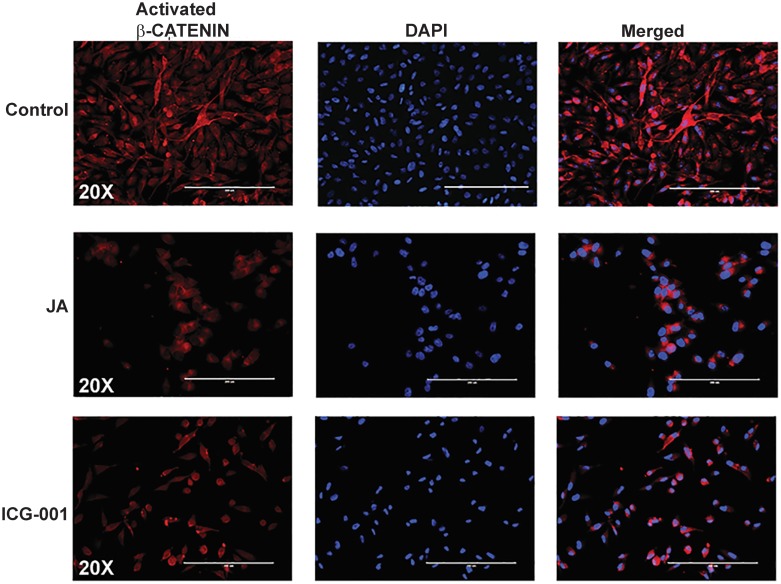
Immunofluorescence (IF) of non-phosphorylated β-catenin protein (amino acids Ser37; Thr41), which is commonly referred to as transcriptionally active β-catenin (ABC). IF of ABC in vehicle control (1% DMSO) **(A)**, JA (B) and ICG-001**(C)** treated MDA-MB-231 cells. Cells were counterstained with DAPI (Blue).

To better understand the molecular mechanisms responsible for the effects of JA against Wnt/β-catenin signaling, we analyzed by immunofluorescence (IF) the levels of non-phosphorylated β-catenin protein (amino acids Ser37; Thr41), which is commonly referred to as transcriptionally active β-catenin (i.e. ABC, Fig 4) [23]. Whereas vehicle-treated (DMSO) cells show ubiquitous amounts of both nuclear and cytoplasmic ABC protein, the JA treated cells have excluded nuclear β-catenin, and ABC is predominantly restricted to the cytoplasm. ICG-001 treatment serves as our control, in which only some cells could be detected with nuclear localization of ABC. These results are consistent with our previous results (Fig 1C, 1E and 1F) that JA inhibits Wnt/β-catenin signaling at the level between the receptor complex and β-catenin activation, thus blocking translocation of β-catenin into the nucleus in order to activate Wnt-responsive elements (WRE) and direct Wnt canonical gene targets.

**Fig 5:**
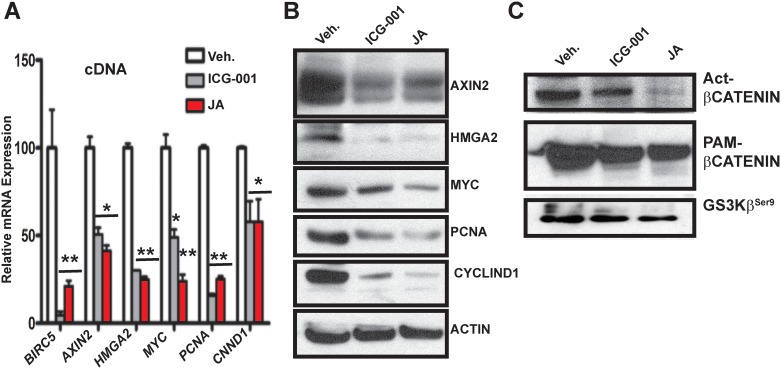
JA inhibits expression on Wnt/β-catenin direct-target genes and degrades non-phosphorylated activated β-catenin protein levels. **(A)** qPCR for *BIRC5*, *AXIN2*, *HMGA2*, *CNND1*, *MYC* and *PCNA* in ICG-001 and JA treated MDA-MB-231 cells. **(B)** Immunoblot analysis for AXIN2, HMGA2, CYCLIND1, PCNA and MYC. ACTIN serves as the loading control. **(C)** Immunoblot analysis for non-phosphorylated activated β-CATENIN (ABC), total pan-β-CATENIN and GS3Kβ^Ser9^ from the same cells and extracts as in panel A and B. Quantification of triplicates using *t-test* p values are ***-p < 0.001, **-p < 0.01 and *p < 0.05 vs. control.

Next, we wanted to determine if exclusion of nuclear β-catenin by JA affects expression of Wnt canonical gene targets by qPCR (Fig 5A). The best known direct target of transcriptionally ABC is AXIN2 (Wnt home page, http://web.stanford.edu/group/nusselab/cgi-bin/wnt/). JA exposure decreases the expression of *AXIN2* mRNA by greater than 50%. Consequently, we also observe significant loss of mRNA expression for *HMGA2*, *MYC*, *PCNA and CYCLIN D1*. Both survivin *(BIRC5*) and ICG-001 exposure function as controls, as we have previously shown that these direct Wnt/β-catenin targets are downregulated by ICG-001 exposure in MDA-MB-231 cells [7]. To determine if the loss of mRNA expression coincided with the loss of protein expression, we conducted immunoblotting (IB) for AXIN2, HMGA2, MYC, PCNA and CYCLIN D1 (Fig 5B), ACTIN functions as our loading control. The loss of protein expression for all five markers is consistent with the loss of mRNA expression. To gain further insights to the effects of JA on transcriptional on ABC protein levels, we directly compared the levels of the non-phosphorylated β-catenin to levels of total β-catenin (i.e. pam-β-catenin antibody; Fig 5C). JA has little effect on total-β-catenin protein levels, but almost completely abolishes the levels of non-phosphorylated β-catenin. Canonical Wnt signaling (ABC) requires inhibition of GSK-3β kinase-activity and phosphorylation at Ser9 of GSK-3β is an indirect read-out for loss of its kinase activity; therefore, we immunoblotted for GSK-3β^Ser9^ in those same samples and determined that expression was largely unchanged. These results suggest that the destruction complex that degrades β-catenin protein levels, which is mediated by AXIN1/APC/GSK-3β, was not activated by JA treatment [24–29].

### Effects of JA on the migration of MDA-MB-231 cells

**Fig 6:**
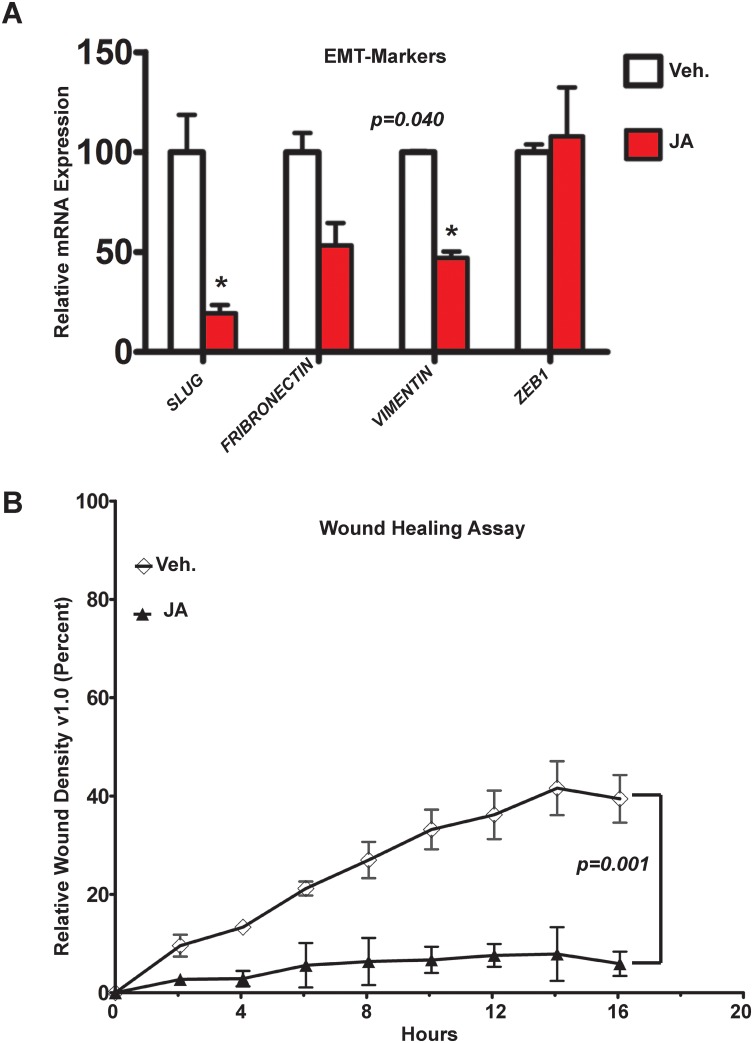
JA inhibits EMT-markers and prevents cell migration in wound healing assays. **(A)** qPCR for *SLUG*, *FIBRONETIN*, *VIMENTIN* and *ZEB1* in ICG-001 and JA treated MDA-MB-231 cells. Results are expressed as mean ± SE, *n* = 3 *p<0.05. **(B)** The number of cells migrated into the scratched area was photographed (340) and calculated as a percentage of migration for 16 hours post treatment. Quantification of triplicates using *t-test*: ***-p < 0.001 vs. control.

Biological activity assays, such as wound healing, offer a quick and effective evaluation of a lead compound (such as JA) to identify undesirable side effects sooner in the drug discovery screening process, rather than later in that process. It is well known that the epithelial to mesenchymal transition (EMT) is required for efficient wound closure in “scratch” assays. Therefore, we sought to determine if JA had any effect on EMT markers and/or the rate of wound closure in wound healing assay (Fig 6A and 6B). We found that JA reduces mRNA expression levels for the classic EMT markers: *SLUG*, *FIBRONECTIN* and *VIMENTIN*, but not for *ZEB1*. Moreover, in MDA-MB-231 cells, 16 hours of exposure to JA (2μM) significantly reduced the ability of cells to migrate (*p = 0*.*001*). The videos for these assays are shown (S1 Video Ctr. and S2 Video JA). These results suggested that JA exhibited anti-invasive behavior toward TNBC at a non-cytotoxic time point, in part through repression of expression of markers that promote EMT.

## Discussion

Triple-negative breast cancer [ER-, PR- and HER2-, (TNBC)] is a highly aggressive breast cancer subtype with a poorer prognosis than other breast cancer subtypes. Unlike other breast cancer subtypes such as, ER+, PR+ and or HER2+, therapeutic options for the TNBC patients are limited due to the lack of identification of pathway-specific targets [30,31]. Additionally, the absence of a validated targeted-therapy further compounds the rise of chemoresistant TNBC as they initial respond to SOC in the neoadjuvant setting but within a short three year period TNBC recur and patients quickly die from metastatic disease [32]. All of these factors lead to an unmet medical need for novel targets to combat chemoresistant metastatic TNBC.

Natural product sources continue to provide front-line pharmacotherapy for millions of people worldwide [33] and our group has previously leveraged this approach to identify natural products that preferentially inhibited proliferation of triple-negative MDA-MB-231 cells over estrogen receptor-positive cells (i.e. abietanes) [9]. Several studies have documented the anti-cancer activity of Jatrophone (JA), a macrocyclic diterpene compound isolated from the *Jatropha isabelli*, on UT51 glioma cells, NCI-ADR/RES drug-resistant ovarian cancer cells and K562 myelogenous leukemia cell model, illustrating anti-proliferative properties [34,35]. In a high throughput phenotypic screen, JA was identified as a promising hit against Raji cells, a Non- Hodgkin Lymphoma, which is a highly invasiveness phenotype. This cellular model has an upregulated Wnt/β-catenin signaling and it is WNT-dependent (via the canonical signaling pathway) [10]. Thus, we hypothesized that JA may be able to inhibit TNBC models that are characterized by upregulated β-catenin signaling. Mechanistically, inhibition of the Wnt reporter TOPFLASH would provide information regarding the mode of action. For the first time, we have shown that JA inhibits TOPFLASH (Wnt/β-catenin signaling) at the level between the receptor complex and β-catenin protein activation.

Women at highest risk for developing metastatic TNBC disease are often young African-Americans (AA) and BRCA mutation carrier women [1,36]. In Memphis, TN, where the AA population is greater than 68%, AA breast cancer is an epidemic, in particular for TNBC diagnoses, providing additional evidence of the breast cancer disparity gap between AA and non-Hispanic white women [37]. We directly tested the effects of JA on proliferation in two different subtypes of TNBC cell lines (i.e. MSL vs. BL1). In the more aggressive MSL-TNBC subtype, MDA-MB-157 cells exhibited lesser sensitivity to JA (2-fold) than the MDA-MB-231 cells. These differences were not observed for the BL1-TNBC subtypes, as could be expected since these subtype are not known to be hyperactive for WNT signaling [6]. Moreover, we were able to confirm that in cell lines derived from TNBC PDX-models obtained from the Huntsman Cancer Institute [20] that the naïve TNBC model HCI-2 responded to JA robustly at an IC_50_ of 1 μM, whereas the highly chemoresistant cell TNBC model, HCI-10, was ~6 times less sensitive to JA exposure.

Moreover, we hypothesized that the differential IC_50_ (DIC_50_) values observed among the various TNBC models was due to the increased expression of mRNA for the *WNT10B* ligand. We confirmed this hypothesis in both the MDA-MB-157 cells and in the highly chemoresistant HCI-10 cells, which both had the highest levels of *WNT10B* mRNA expression with the highest DIC_50_ values as compared to the other TNBC cells lines tested. Additional confirmation of these results was shown in HeLa cervical cancer cells and in osteosarcoma U2OS cells that expressed the highest mRNA levels for *WNT10B* expression than in the control *WNT10B* low and/or negative cell lines, including MCF-7 (ER+), MCF-10A (TNBC control “normal”) or MG63 (osteosarcoma).

To further confirm our findings, immunoblot analysis of the β–catenin pathway was conducted. By directly comparing non-phosphorylated β-catenin protein levels at serine 37 (Ser37) and threonine 41 (Thr41) to total β-catenin [23], we demonstrated that JA was able to uncouple translocation of β-catenin to the cytoplasm without impacting the turnover of total β-catenin protein levels. The consequence of loss of transcriptional activity mediated by β–catenin direct targets genes included reduced expression of the genes *AXIN2*, *HMGA2*, *MYC* and *CCND1*. Moreover, JA was capable of inhibiting MDA-MB-231 cell migration at a time point prior to observation of its cytotoxic, anti-proliferative effects. The wound healing assays provide us early proof of principle evidence that JA has a favorable anti-cancer profile to justify screening for its ability to inhibit or to prevent metastatic disease *in vivo*.

## Conclusion

In summary, we have characterized the anti-proliferative effects of the natural compound Jatrophone in a TNBC cells and in treatment naïve and chemoresistant TNBC PDX cells. This study provides compelling evidence that JA exerts its anti-proliferative and anti-migratory properties *via* interfering with Wnt/β-catenin signaling by exclusion of nuclear ABC and repression of downstream Wnt target genes. JA also exerts potent repression of cell migration in part through reduction of genes necessary to induce EMT, which is required for wound healing. In total, our observations strongly suggest that Jatrophone is poised to be further developed as a lead molecule to combat highly chemoresistant metastatic TNBC.

## Supporting information

S1 Fig WST-1 proliferation assays for 48 hours utilizing JA at various dosages. **A)** The calculated DIC_50_ for MDA-MB-231, MDA-MB-157, HCC-38, MDA-MB-468, HCl-2 and HCl-10 cell lines. **B)** The mRNA expression for *WNT10B* in MCF-7, MCF-10A, MDA-MB-231, MDA-MB-157, HCC-38, MDA-MB-468, Hcl-2, HCl-10, HeLa, U2OS and MG63 cells. Relative to 18S expression. **C)** Ovarian cancer HeLa cells JA dosages ranging from 100 nM-30 μM and **(D)** Osteosarcoma cell lines U2OS and MG63 ranging from 1–5 μM. The IC_50_ of JA HeLa cells was determined by n = 3, in triplicate, using the two-tailed *t-test p*-values were calculated: *** < 0.001 relative to vehicle control cells (DMSO). Osteosarcoma results are expressed as mean ± SE, *n* = 2.
(TIF)

S1 Table Primer sequences used for qPCR. (PDF)

S1 Video Wound healing assay in vehicle treated MDA-MB-231 cells. (AVI)

S2 Video Wound healing assay in JA treated MDA-MB-231 cells. (AVI)
