# Correction: The natural compound Jatrophone interferes with Wnt/β-catenin signaling and inhibits proliferation and EMT in human triple-negative breast cancer

**DOI:** 10.1371/journal.pone.0197796

**Published:** 2018-05-17

**Authors:** Iram Fatima, Ikbale El-Ayachi, Ling Taotao, M. Angeles Lillo, Raisa I. Krutilina, Tiffany N. Seagroves, Tomasz W. Radaszkiewicz, Miroslav Hutnan, Vitezslav Bryja, Susan A. Krum, Fatima Rivas, Gustavo A. Miranda-Carboni

The fifth author’s name is spelled incorrectly. The correct name is: Raisa I. Krutilina. The correct citation is: Fatima I, El-Ayachi I, Taotao L, Lillo MA, Krutilina RI, Seagroves TN, et al. (2017) The natural compound Jatrophone interferes with Wnt/β-catenin signaling and inhibits proliferation and EMT in human triple-negative breast cancer. PLoS ONE 12(12): e0189864. https://doi.org/10.1371/journal.pone.0189864
