# Supplementary figures and images for: The natural compound Jatrophone interferes with Wnt/β-catenin signaling and inhibits proliferation and EMT in human triple-negative breast cancer
Source: PLoS One. 2017 Dec 27;12(12):e0189864. doi: 10.1371/journal.pone.0189864 (PMC5744972; doi:10.1371/journal.pone.0189864)

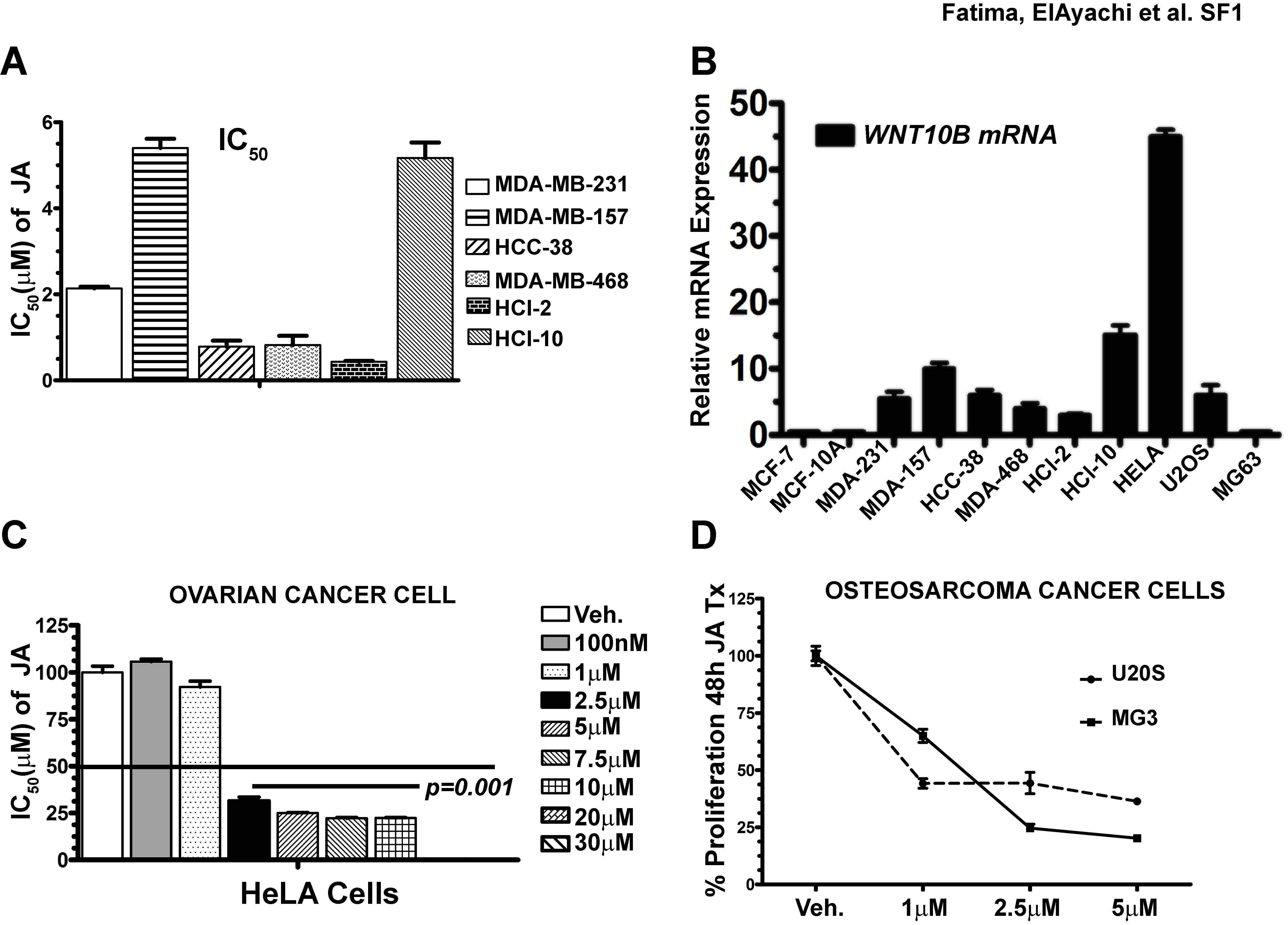

Supplement: S1 Fig — A) The calculated DIC50 for MDA-MB-231, MDA-MB-157, HCC-38, MDA-MB-468, HCl-2 and HCl-10 cell lines. B) The mRNA expression for WNT10B in MCF-7, MCF-10A, MDA-MB-231, MDA-MB-157, HCC-38, MDA-MB-468, Hcl-2, HCl-10, HeLa, U2OS and MG63 cells. Relative to 18S expression. C) Ovarian cancer HeLa cells JA dosages ranging from 100 nM-30 μM and (D) Osteosarcoma cell lines U2OS and MG63 ranging from 1–5 μM. The IC50 of JA HeLa cells was determined by n = 3, in triplicate, using the two-tailed t-test p-values were calculated: *** < 0.001 relative to vehicle control cells (DMSO). Osteosarcoma results are expressed as mean ± SE, n = 2. (TIF) [file pone.0189864.s001.tif]
