# Supplementary material for: The natural compound Jatrophone interferes with Wnt/β-catenin signaling and inhibits proliferation and EMT in human triple-negative breast cancer
Source: PLoS One. 2017 Dec 27;12(12):e0189864. doi: 10.1371/journal.pone.0189864 (PMC5744972; doi:10.1371/journal.pone.0189864)
Supplement: S1 Table — (PDF) [file pone.0189864.s002.pdf]

Suppl. Information:

Table 1. Primer sequences used for qPCR:

Primer	Sequence	Orientation
hWNT10B	5’-TGG GAT GTG TAG CCT TCT CC-3’	S
	5’-CCC AGC CAA AAG GAG TAT GA-3’	AS
hCDK4	5’-TCA TGC CAA TTG CAT CGT TCA CCG -3’	S
	5’-ACA GGT GTT GCA TAT GTG GAC TGC -3’	AS
hCCND1	5’-CAG AAG TGC GAA GAG GAG GTC-3’	S
	5’-TCA TCT TAG AGG CCA CGA ACA T-3’	AS
hCCNE1	5’-TTA CCC AAA CTC AAC GTG CAA GCC-3’	S
	5’- AGA GGG TGT TGC TCA AGA AAG TGC-3’	AS
hCCNA1	5’- TGT CTG TTC TGA GAG GGA AAC TGC -3’	S
	5’- AAG GAG AAA CTG GTT GGT GGT TGG -3’	AS
hCCNB1	5’- ATT GTG TGC CCA AGA AGA TGC TGC -3’	S
	5’- TTA GAT GCT CTC CGA AGG AAG TGC-3’	AS
hBIRC5	5’- CCG CAT CTC TAC ATT CAA GAA CTG GC	S
	5’- TTG ACA GAA AGG AAA GCG CAA CCG -	AS
hAXIN2	5’-TCA AGT GCA AAC TTT CGC CAA CCG-3’	S
	5’-TGG TGC AAA GAC ATA GCC AGA ACC-3’	AS
hMYC	5’-TCT CCA CAC ATC AGC ACA ACT ACG-3’	S
	5’-TGT GTT CGC CTC TTG ACA TTC TCC-3’	AS
hHMGA2	5’-TCCGTGCCCGACCCTAT-3’	S
	5’-CCTTGAAATGTTAGGCGGGG -3’	AS
hPCNA	5’- TGT AAA CCT GCA GAG CAT GGA CTC G-	S
	5’- AAA TAC TAG CGC CAA GGT ATC CGC-3’	AS
hSLUG	5’- TTT CTG GGC TGG CCA AAC ATA AGC -3’	S
	5’- AAT GTG TCC TTG AAG CAA CCA GGG -3’	AS
hFIBRONECTIN	5’- TGT CAA CGA AGG CTT GAA CCA ACC -3’	S
	5’- AGT TCA CAC CAT TGT CAT GGC ACC -3’	AS
hVIMENTIN	5’-ATG AAA GTG TGG CTG CCA AGA ACC-3’	S
	5’-TCT CTT CCA TTT CAC GCA TCT GGC-3’	AS
hZEB1	5’-ATG CAC AAC CAA GTG CAG AAG AGC-3’	S
	5’-AGG CTG ATC ATT GTT CTT GGC AGG-3’	AS
h18S	5’- CCG CGG TTC TAT TTT GTT GGT -3’	S
	5’- CTC TAG CGG CGC AAT ACG A -3’	AS

Legend: Antisense (AS), Sense (S), and human (h).
